# Supplementary material for: Effect of Transcutaneous Auricular Vagus Nerve Stimulation in Chronic Low Back Pain: A Pilot Study
Source: J Clin Med. 2024 Dec 13;13(24):7601. doi: 10.3390/jcm13247601 (PMC11677670; doi:10.3390/jcm13247601)
Supplement: Supplementary file 1 [file jcm-13-07601-s001.zip › Supplementary Table S1.pdf]

Supplementary Table S1: Detailed description of the main characteristics of the questionnaires used in the VALOM study

Questionnaire	Description	Clinical Cutoff Values	Key References
**Oswestry Disability Index (ODI)**	Evaluates disability levels in individuals with low back pain. It quantifies the impact of back pain on daily life through 10 domains, including pain intensity, personal care, lifting, walking, and social life. It is one of the most widely used measures for back pain-related disability in clinical and research settings. It ranges from 0 to 100%.	0–20%: Minimal disability; 21–40%: Moderate; 41–60%: Severe; 61–80%: Crippled; 81–100%: Bed-bound.	<u>*English reference:*</u><br>Fairbank JC, et al. The Oswestry low back pain disability questionnaire. Physiotherapy, 1980. DOI: 10.1097/00007632-198003000-00009.<br><br><u>*French reference:*</u><br>Vogler D, et al. Cross-cultural validation of the Oswestry disability index in French. Annales de Réadaptation et de Médecine Physique, 2008. DOI: 10.1016/j.annrmp.2008.03.006.
**EQ-5D-5L**	Measures health-related quality of life (HRQoL) across five dimensions: mobility, self-care, usual activities, pain/discomfort, and anxiety/depression. The 5-level version increases sensitivity by adding more gradations to each dimension. The tool provides an overall health index value based on population tariffs and a visual analog scale (VAS) for subjective health evaluation. This score ranges from -0.594 (worst health) to 1 (perfect health) and the VAS from 0 to 100.	EQ-5D-5L VAS mean value in a French sample: 79.8/100	<u>*English reference:*</u><br>Herdman, M., et al. Development and preliminary testing of the new five-level version of EQ-5D (EQ-5D-5L). Qual Life Res, 2011. DOI: 10.1007/s11136-011-9903-x.<br><br><u>*French references:*</u><br>Chevalier, J. Mesure de l'utilité Attachée Aux États de Santé : Valorisation de l'index d'utilité EQ-5D et Évolution de l'échelle Actuelle En France. These de doctorat, Paris 9, 2010.<br>Andrade, L.F. et al. A French Value Set for the EQ-5D-5L. Pharmacoeconomics 2020. DOI: 10.1007/s40273-019-00876-4.
**Hospital Anxiety and Depression Scale (HADS)**	Screens for anxiety and depression symptoms in medical patients. It is composed of two subscales (anxiety and depression), each with 7 items, leading to 2 subscores ranging from 0 to 21. The tool excludes somatic symptoms to	Anxiety/Depression Subscales: 0–7 (Normal); 8–10 (Borderline); ≥11 (Clinically significant).	<u>*English Reference:*</u><br>Zigmond AS, et al. The hospital anxiety and depression scale. Acta Psychiatrica Scandinavica, 1983. DOI: 10.1111/j.1600-0447.1983.tb09716.x.

	prevent confounding with physical illnesses. It demonstrates high reliability and accuracy and is widely used in general medical and psychiatric settings.		*French Reference:*<br>Lépine, JP et al. Evaluation of anxiety and depression among patients hospitalized on an internal medicine service. Ann Med Psychol (Paris) 1985. PMID: 4037594
**Pain Catastrophising Scale (PCS)**	Assesses exaggerated and negative cognitive and emotional responses to pain. It measures rumination, magnification, and helplessness. It is highly reliable and sensitive, making it applicable for clinical and research use. PCS ranges from 0 to 52. High PCS scores are associated with greater psychological distress and impaired pain management. It is widely used in pain research and clinical settings.	Total PCS ≥30 indicates clinically significant catastrophising	*English Reference:*<br>Sullivan MJL, et al. Psychological Assessment, 1995. DOI: 10.1037/1040-3590.7.4.524.<br>*French Reference:*<br>French DJ, et al. PCS-CF: A French-language, French-Canadian adaptation of the Pain Catastrophizing Scale. Canadian Journal of Behavioural Science, 2005. DOI : 10.1037/h0087255.
