# Supplementary material for: Effect of Transcutaneous Auricular Vagus Nerve Stimulation in Chronic Low Back Pain: A Pilot Study
Source: J Clin Med. 2024 Dec 13;13(24):7601. doi: 10.3390/jcm13247601 (PMC11677670; doi:10.3390/jcm13247601)
Supplement: Supplementary file 1 [file jcm-13-07601-s001.zip › Supplementary Table S2.pdf]

Supplementary Table S2: Evolution of low back pain VAS at any timepoint: additional analysis with a linear mixed model.

**Variable**	**Effect**	**Evolution from baseline**	**Std Error**	**p-value at any time**	**Global p-value**
Time	Week 1	-12.78	4.79	0.009	< 0.001
	Week 2	-17.13	4.90	< 0.001	
	Week 3	-18.00	4.79	< 0.001	
	1 month	-16.28	4.79	< 0.001	
	3 months	-24.49	4.96	< 0.001	

As an additional analysis, a linear mixed model was performed with a fixed time effect and a random subject effect. There was a significant effect of time (p-global < 0.001). The difference in VAS compared with inclusion was significant at all time points considered (all p-values at each time point were less than 0.05).
