# Supplementary material for: Effect of Transcutaneous Auricular Vagus Nerve Stimulation in Chronic Low Back Pain: A Pilot Study
Source: J Clin Med. 2024 Dec 13;13(24):7601. doi: 10.3390/jcm13247601 (PMC11677670; doi:10.3390/jcm13247601)
Supplement: Supplementary file 1 [file jcm-13-07601-s001.zip › Supplementary Table S3.pdf]

Supplementary Table S3: Baseline characteristics comparison of responders and non-responders

Characteristics at baseline		Non-responders	Responders	p-value
Sex		n=13	n=14	0.25
	Women	9 (69.2)	6 (42.9)	
	Men	4 (30.8)	8 (57.1)	
Age (years)		n=13	n=14	0.66
	Mean (SD)	49.1 (15.5)	47.1 (13.3)	
Work situation: work stoppage		n=7	n=7	
	No	5 (71.4)	5 (71.4)	
	Yes	2 (28.6)	2 (28.6)	
BMI (kg/m²)		n=13	n=14	0.44
	Mean (SD)	24.6 (4.6)	26.6 (5.4)	
Low back pain VAS (/100)		n=13	n=14	0.54
	Mean (SD)	60.4 (14.2)	65.4 (13.7)	
Current analgesics consumption, n (%)		n=13	n=14	0.45
	No	8 (61.5)	6 (42.9)	
	Yes	5 (38.5)	8 (57.1)	
Weekly cigarettes consumption		n=13	n=14	0.88
	Mean (SD)	15 (39.6)	10.4 (20.1)	
Coffee consumption (cups / week)		n=13	n=14	0.25
	Mean (SD)	8.9 (10.8)	19 (19.9)	
Tea consumption (cups / week)		n=13	n=14	0.42
	Mean (SD)	2.2 (4.4)	0.6 (1.9)	
Physical activity practice		n=13	n=14	0.69
	No	4 (30.8)	6 (42.9)	
	Yes	9 (69.2)	8 (57.1)	
Duration of pain (months)		n=13	n=14	0.80
	Mean (SD)	157.9 (137.8)	131.7 (99.9)	
History of analgesics consumption		n=13	n=14	
	Yes	13 (100)	14 (100)	
History of opioids consumption		n=13	n=14	1.00
	No	6 (46.1)	7 (50)	
	Yes	7 (53.9)	7 (50)	
History of infiltration		n=13	n=14	**0.02**
	No	3 (23.1)	10 (71.4)	
	Yes	10 (76.9)	4 (28.6)	
History of lumbar surgery		n=13	n=14	0.16
	No	9 (69.2)	13 (92.9)	
	Yes	4 (30.8)	1 (7.1)	
ODI (/100)		n=13	n=14	0.14
	Mean (SD)	36.4 (10.9)	31.4 (13.6)	
EQ-5D-5L VAS (/100)		n=13	n=13	0.91
	Mean (SD)	52.7 (15.1)	55.1 (19.6)	
HADS total score (/42)		n=13	n=14	0.07
	Mean (SD)	20.2 (4.8)	16.1 (6.4)	
PCS total score (/52)		n=12	n=13	**0.02**
	Mean (SD)	31.4 (9.5)	21.6 (11.5)	
