# Supplementary material for: Effect of Transcutaneous Auricular Vagus Nerve Stimulation in Chronic Low Back Pain: A Pilot Study
Source: J Clin Med. 2024 Dec 13;13(24):7601. doi: 10.3390/jcm13247601 (PMC11677670; doi:10.3390/jcm13247601)
Supplement: Supplementary file 1 [file jcm-13-07601-s001.zip › Supplementary Table S4.pdf]

Supplementary Table S4: Changes in HRV parameters after 1 and 3 months of using taVNS

	Mean evolution at 1 month (SD)<br>n = 27	p-value	Mean evolution at 3 months (SD)<br>n = 24	p-value
RMSSD, ms	-12.7 (43.7)	0.23	-3.7 (75.2)	0.05
RRI, ms	-32.9 (94.6)	0.08	-46.7 (80.4)	0.01
SSDN, ms	-11.1 (34.6)	0.28	-3.5 (54.2)	0.29
HF power, ms^2	-805.3 (3175.6)	0.30	632 (8839.4)	0.07
LF/HF ratio	0.2 (2.7)	0.82	0.5 (2.7)	0.18

RRI: R-R intervals
RMSSD: Root Mean Square of Successive Differences of RRI
SSDN: Standard Deviation of the NN interval
HF power: high frequency power
LF / HF ratio: low frequency / high frequency ratio
